# Singlet oxygen damages the function of Photosystem II in isolated thylakoids and in the green alga *Chlorella sorokiniana*

**DOI:** 10.1007/s11120-021-00841-3

**Published:** 2021-05-19

**Authors:** Faiza Bashir, Ateeq Ur Rehman, Milán Szabó, Imre Vass

**Affiliations:** 1 Biological Research Centre, Institute of Plant Biology, Eötvös Loránd Research Network (ELKH), Szeged, Hungary; 2 Doctoral School of Biology, University of Szeged, Szeged, Hungary; 3 Climate Change Cluster, University of Technology Sydney, Sydney, Australia

**Keywords:** Singlet oxygen, Chlorophyll fluorescence imaging, Photoinhibition, Thylakoid membranes, Microalgae

## Abstract

Singlet oxygen (^1^O_2_) is an important damaging agent, which is produced during illumination by the interaction of the triplet excited state pigment molecules with molecular oxygen. In cells of photosynthetic organisms ^1^O_2_ is formed primarily in chlorophyll containing complexes, and damages pigments, lipids, proteins and other cellular constituents in their environment. A useful approach to study the physiological role of ^1^O_2_ is the utilization of external photosensitizers. In the present study, we employed a multiwell plate-based screening method in combination with chlorophyll fluorescence imaging to characterize the effect of externally produced ^1^O_2_ on the photosynthetic activity of isolated thylakoid membranes and intact *Chlorella sorokiniana* cells. The results show that the external ^1^O_2_ produced by the photosensitization reactions of Rose Bengal damages Photosystem II both in isolated thylakoid membranes and in intact cells in a concentration dependent manner indicating that ^1^O_2_ plays a significant role in photodamage of Photosystem II.

## Introduction

Photosynthesis is a basic process in the biosphere in which green plants, algae and cyanobacteria utilize solar energy to synthesize carbohydrates from carbon dioxide and water see (Renger and Govindjee 1985). The initial, light-dependent steps of the photosynthetic process are performed by four protein complexes embedded in the thylakoid membrane, namely Photosystem II (PSII), Photosystem I (PSI), cytochrome b/_6_ f complex and the ATP synthase. NADPH and ATP, which are produced in the light-dependent reactions are utilized in the so-called dark reactions of photosynthesis, which leads to the fixation of atmospheric CO_2_ and result in the production of carbohydrates.

Although light is the ultimate driving force of photosynthesis, light at the same time is also a significant damaging agent, which can impair photosynthetic activity. This process is called photoinhibition, whose mechanism remains controversial despite intensive research during the last decades (Jones and Kok 1966; Ohad et al. 1984; Barber and Andersson 1992; Aro et al. 1993; Nishiyama et al. 2006; Vass and Cser 2009; Oguchi et al. 2009; Murata et al. 2012; Tyystjärvi 2013; Zavafer et al. 2017, 2019). The most light-sensitive site in the photosynthetic apparatus is the PSII complex in which electron transport activity is impaired and the D1 (and D2) reaction centre protein is degraded, see (Aro et al. 1993). In addition to PSII, PSI (Sonoike and Terashima 1994; Sonoike et al. 1995; Suorsa et al. 2012; Tiwari et al. 2016; Lima-Melo et al. 2019), as well as the Chl containing light harvesting antenna structures can also be damaged by light (Zolla and Rinalducci 2002; Rinalducci et al. 2004, 2008; Lingvay et al. 2020). The detrimental effects of light linearly depend on light intensity (Tyystjarvi and Aro 1996) and can occur at all light intensities (Keren et al. 1997; Kou et al. 2012), therefore plants have evolved a protective repair mechanism, by which light induced loss of photosynthetic activity can be restored. This repair mechanism proceeds via de novo synthesis of the light-damaged D1 (and D2) subunit(s) of PSII (Aro et al. 1993; Nixon et al. 2010; Komenda et al. 2012; Murata and Nishiyama 2018; Li et al. 2018). Due to competing photodamage and repair processes net loss of PSII activity occurs when the rate of photodamage exceeds the rate of repair.

Although the exact mechanism of photodamage and its repair is not yet fully clarified there is a consensus in the literature that reactive oxygen species (ROS) are involved in the overall photoinhibition process (Barber and Andersson 1992; Anderson and Chow 2002; Nishiyama et al. 2006; Krieger-Liszkay et al. 2008; Pospísil 2009, 2012; Vass 2011; Fischer et al. 2013).

Singlet oxygen (^1^O_2_), which is one of the most important ROS, is an excited state of molecular oxygen which is highly reactive (Ogilby 2010). It damages proteins, lipids and nucleic acids, so it is an important reactive oxygen species (ROS) in biological systems. On the other hand ^1^O_2_ is also an important signaling molecule (Triantaphylidès and Havaux 2009). It is less stable than triplet oxygen (^3^O_2_), and may be formed in a number of ways; however, the typical way is by energy transfer from the triplet state of a photosensitized pigment or dye molecule (Hirakawa et al. 2011; Fischer et al. 2013). Production of ^1^O_2_ has been demonstrated in isolated photosynthetic complexes (Macpherson et al. 1993; Hideg et al. 1994a, b; Telfer et al. 1999) and intact photosynthetic systems (Hideg et al. 1998; Flors et al. 2006; Rehman et al. 2013, 2016b). It has also been shown that photolerance in highly light tolerant algal species is accompanied by decreased ^1^O_2_ production (Treves et al. 2016; Virtanen et al. 2021). However, the exact sites and mechanisms of ROS action in photoinhibition are debated. According to one idea ROS, including ^1^O_2_, affects only the repair of PSII by inhibiting translation elongation of the *psbA* gene, thereby preventing de novo synthesis of photodamaged D1 protein in the cyanobacterium *Synechocystis* PCC 6803 (Nishiyama et al. 2001, 2004, 2006). Recent results however, have shown that ^1^O_2_ does not affect the PSII repair process in green algae (Dall’Osto et al. 2019; Barera et al. 2021). Moreover, it has also been proposed that β-carotene molecules, which are oxidized by ^1^O_2_ act as signaling agents that induce defense mechanisms that alleviate the effects of PSII photodamage conditions (D'Alessandro and Havaux 2019). Other studies have demonstrated a clear correlation between the rate of ^1^O_2_ formation and the rate of PSII photodamage in various experimental systems (Rehman et al. 2013; Hakkila et al. 2013, 2014; Bersanini et al. 2014; Sedoud et al. 2014; Treves et al. 2016) pointing to the role of ^1^O_2_ as an agent that damages directly the function and structure of the PSII complex (Vass and Cser 2009).

One useful approach to assess to role of ^1^O_2_ in PSII photodamage is the generation of ^1^O_2_ by externally added photosensitizers. A previous study has compared the ability of different ^1^O_2_ photosenzitizers (Rose Bengal, Methylene Violet, Neutral Red and Indigo Carmine) to penetrate into plant cells (Kovács et al. 2014). It was concluded that Rose Bengal (RB), which is localized in the chloroplasts and can be excited by green light with only minor effect on photosynthetic electron transport, was the most efficient ^1^O_2_ producing photosensitizer that resulted in PSII activity loss and D1 protein degradation (Kovács et al. 2014). Similar results were obtained earlier also by using RB infiltrated tobacco leaves (Hideg et al. 2007). However, in these studies very high RB concentrations (100 μM, and 1 mM, respectively) had to be used to ensure infiltration into the tobacco leaf, and there was no information about the actual level of ^1^O_2_ inside the cells and in the thylakoid membranes. Therefore, the possibility that unnaturally high ^1^O_2_ levels caused unspecific PSII damage in these plant studies could not be excluded. It is of note that ^1^O_2_ production in the presence of low micromolar RB concentrations has been shown to induce growth retardation and specific gene expression in the green alga *Chlamydomonas reinhardtii* (Leisinger et al. 2001; Fischer et al. 2004), however, direct PSII damage was not demonstrated in these studies.

In the present study we investigated the effect of ^1^O_2_, when produced by low concentrations of RB, on the photosynthetic activity of isolated spinach thylakoids and intact *Chlorella sorokiniana* cells. Our data show a RB concentration-dependent loss of PSII activity in both systems (in the 1–10 μM RB range), which confirm that externally generated ^1^O_2_ can directly inactivate the PSII complex.

## Material and methods

### Biological materials

*Chlorella sorokiniana* cells (originating from Culture collection of Autotrophic Organisms (CCALA, Trebon, Czech Republic) were propagated in BG-11 growth medium (pH 7.5) and grown at 24 ℃ at the irradiance of 60–70 μmol photons m^−2^ s^−1^ white light, in 500 mL flasks containing 200 mL of BG-11 kept on an orbital shaker. Four days old cultures in the exponential growth phase (A_750_ of 0.2–0.3) were used for the experiments. The chlorophyll concentration (Chl a + b) was determined by a UV-1601 (SHIMADZU) spectrophotometer after extracting the pigments with acetone:DMSO 1:1. Chl a + b content was calculated according to (Shoaf and Lium 1976). Cells were harvested by centrifugation at 6500 g for 5 min and re-suspended in 100 mL of fresh BG-11 medium at concentration of 5 μg of Chl mL^−1^. The cells were kept under normal growth conditions for one hour before measurement.

### Thylakoid membrane preparation

Thylakoid membranes were isolated from fresh spinach leaves as described earlier by (Anderson 1981), and suspended in a buffer solution containing 50 mM tricine (pH = 7.5), 7 mM MgCl_2_, 7 mM CaCl_2_ and 0.3 M Sorbitol. Thylakoid membranes were stored at − 80 °C for further experiments. Before measurements, thylakoid membranes were resuspended in the same buffer with the Chl concentration of 5 μg of Chl mL^−1^.

### Experimental procedure for the Rose Bengal assay

The Rose Bengal (RB) stock solution (500 μM in distilled water) was freshly prepared before each experiment. Samples (in triplicates) were incubated in the presence of 0, 1, 5 and 10 μM RB in 24 well plates (Vision Plate™ 24 Well, 4titude, Brooks Life Sciences, U.K.), which were placed in a temperature controlled incubation chamber (temperature was maintained at 24 ℃ for *Chlorella* cells and at 4 ℃ for isolated thylakoid membranes, using a water circulation heater-chiller, Julabo). The illumination was provided from the top by a LED array using green light (50 µmol photons m^−2^ s^−1^) in combination with white light (190 µmol photons m^−2^ s^−1^, green + white irradiance was 240 µmol photons m^−2^ s^−1^) to excite the RB dye in the 520–550 nm spectral range, without providing excess excitation to the photosynthetic processes. In *Chlorella* cells the light treatment was performed both in the absence and presence of 300 μg mL^−1^ lincomycin, which inhibits protein synthesis dependent PSII repair. At the indicated time points, the well plates containing the samples were transferred for chlorophyll fluorescence imaging. The same experiment was also performed in complete darkness, under identical experimental conditions that were applied for light treatment but without applying the LED illumination.

### Chlorophyll fluorescence imaging and measurements of the maximum quantum yield of PSII

Chlorophyll fluorescence of the samples incubated in the well plates was assessed by pulse-amplitude modulated imaging (PAM) MAXI (Imaging-PAM M-series Chlorophyll Fluorometer Heinz Walz GmbH, Germany). The minimum fluorescence in dark adapted state, F_0_, was measured using a weak measuring light (ML settings = 4, PPFD < 0.3 µmol photons m^−2^ s^−1^) and maximum fluorescence, *F*_m_, was measured by applying a saturation pulse (SP setting = 10, length: 0.8 s, PPFD: approx. 2000 µmol photons m^−2^ s^−1^). The maximum quantum yield of PSII was calculated as *F*_v_/*F*_m_ = (*F*_m_ − *F*_0_)/*F*_m_. Samples were dark-adapted for 5 min before the measurements. The software ImagingWin was applied to select circular areas of interest for individual wells, in which *F*_0_ and *F*_m_ were measured and *F*_v_/*F*_m_ was calculated. The ML intensity was adjusted so that the F_0_ signal did not drop below 0.1, to allow reliable measurements of *F*_0_ and *F*_m_. To ensure homogeneity of the chlorophyll fluorescence signal, an image correction was performed according to the manufacturer, which enabled homogeneous signal intensity across the imaged area. Despite all corrections, some heterogeneity of the incident irradiance in the individual wells always remains, therefore minor spatial variations in the amplitude of *F*_0_ and *F*_m_ cannot be completely avoided. However, this can be compensated by the ratio calculation of maximum quantum yield of PSII (*F*_v_/*F*_m_), which eliminates the spatial heterogeneity of the amplitude of the basic fluorescence parameters (Schreiber et al. 2007). RB in the thylakoid buffer or in the culture medium did not show any autofluorescence with the 450 nm ML and SP light source of the PAM system in the 1–10 μM concentration range, as RB does not absorb light in this spectral region, therefore, it does not interfere with the Chl fluorescence imaging measurements.

### Oxygen evolution/uptake measurements

Oxygen evolution and uptake rate was measured using a 4-channel FireStingO2 (FSO2-4) fiber-optical oxygen meter with optode sensors (Robust oxygen probe, OXROB10) (PyroScience GmbH, Aachen, Germany). Oxygen measurements were performed in 1 × 1 cm plastic cuvettes, which were placed under identical illumination (green-white light, 240 µmol photons m^−2^ s^−1^) and incubation conditions that were applied for the multiwell plate experiments. The cuvettes were mounted 45° onto a plastic platform, to allow homogeneous light penetration to the samples. The optodes were placed into the cuvettes containing the samples and sealed with plastic stoppers. Before measurements, 2-point calibration was performed using air-saturated water and deoxygenated water (with Na_2_S_2_O_4_) according to the manufacturers’ specifications. 2 mL cultures with the Chl content of 5 μg mL^−1^ were loaded into the cuvettes, and measurements in the presence or absence or RB, and in the presence or absence of lincomycin were performed as specified at the relevant sections (‘Results’ and ‘Discussion’). Dark respiration was measured for 5 min in complete darkness, then oxygen evolution/uptake rate (depending on the applied conditions) was measured for 5 min after switching on the light. To determine ^1^O_2_ production by O_2_ uptake, 5 mM Histidine was applied. Oxygen evolution/uptake rates are expressed in μmol O_2_ mg Chl^−1^ h^−1^.

## Statistical analysis

Statistical analysis was performed using OriginPro (OriginLab Corporation, Northampton, MA, USA). One-way analysis of variance (ANOVA) and Tukey’s post-hoc multiple comparison tests (*α* = 0.05) were performed on independent samples to detect statistically significant differences between treatments. Normality tests were performed using Kolmogorov–Smirnov method and the homogeneity of variance test was performed using Levene’s method.

## Results

### Externally produced ^1^O_2_ accelerates photodamage of PSII in isolated thylakoid membranes

**Fig. 1:**
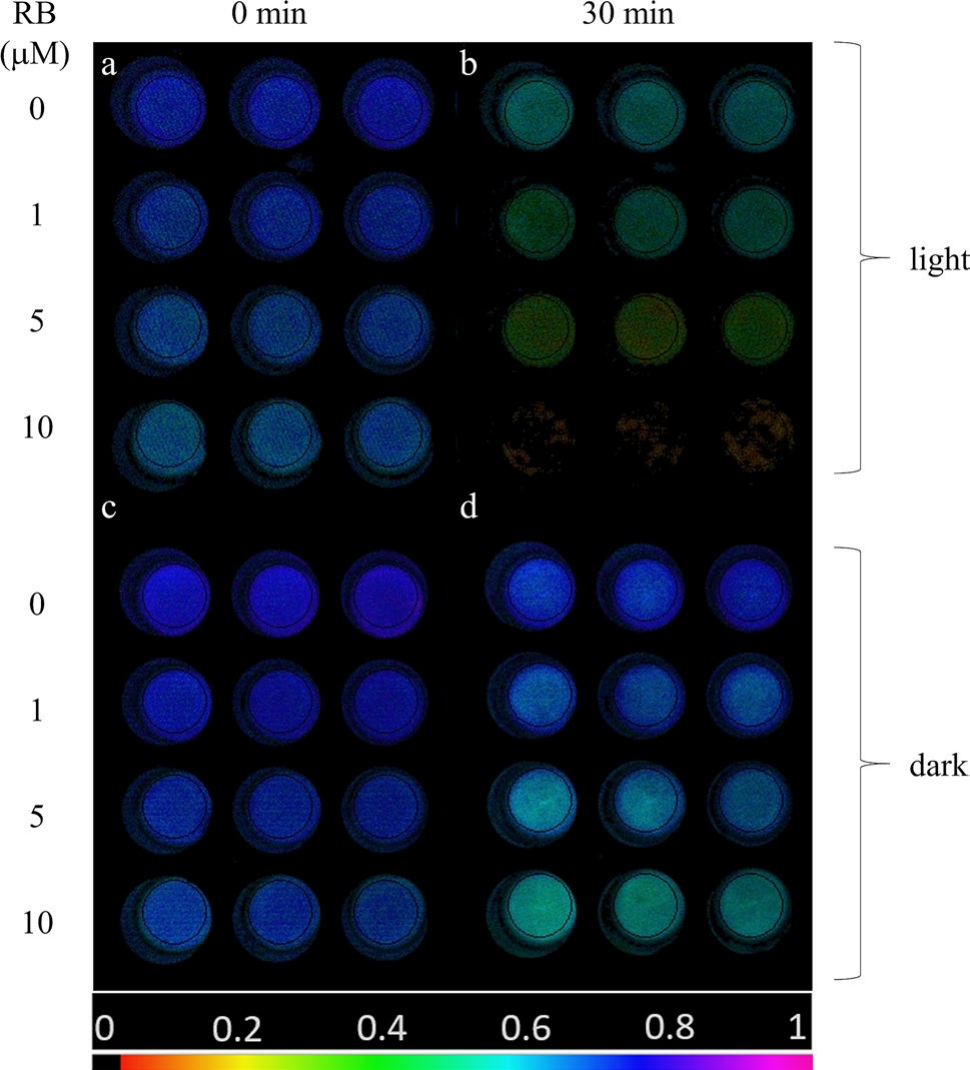
*F*_v_/*F*_m_ image of thylakoid membranes illuminated with green + white light (panels **a**, **b**) or kept in darkness (**c**, **d**) for 0 min (**a**, **c**) and 30 min (**b**, **d**). In all panels first row represents control (0 µM RB), second row 1 µM RB, third row 5 µM RB, fourth row 10 µM RB (*n* = 3). *F*_v_/*F*_m_ values are determined in the selected circular areas of interest. The color bar indicates the relative intensity of *F*_v_/*F*_m_

To investigate the effect of externally generated ^1^O_2_ on photosynthetic activity of isolated thylakoid membranes, samples were illuminated in the presence of various concentrations of RB in 24-well plate and PSII activity was quantified by determining the F_v_/F_m_ values in each well using variable Chl fluorescence imaging. This is a particularly useful approach when PSII activity has to be determined simultaneously in a large number of samples (Schreiber et al. 2007). At the initial time point (*t* = 0), thylakoid membranes imaged in the well plates exhibited homogeneous *F*_v_/*F*_m_ values of approx. 0.66–0.74, however, somewhat lower *F*_v_/*F*_m_ was observed with increasing RB concentrations (Fig. 1a). *F*_v_/*F*_m_ decreased during the light exposure in a RB concentration dependent manner; at 10 μM the *F*_v_/*F*_m_ essentially dropped to 0 after 30 min (Fig. 1b). The *F*_v_/*F*_m_ values also showed some decrease in darkness, which was more pronounced at higher RB concentrations (Fig. 1c, d). These findings demonstrate that illumination of isolated thylakoids in the presence of RB induces the loss of PSII activity.

**Fig. 2:**
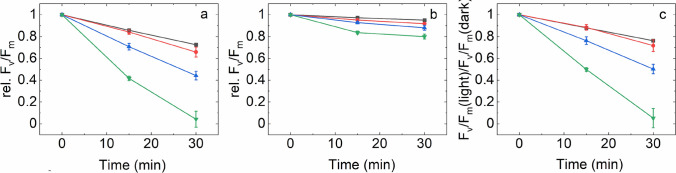
Effect of RB on the maximum quantum efficiency of PSII (*F*_v_/*F*_m_) of thylakoid membranes treated with green-white light (**a**), or kept in dark (**b**) for the indicated time periods. Panel **c** represents the ratio of *F*_v_/*F*_m_ recorded during the light treatment or dark incubation experiment [*F*_v_/*F*_m_ values in panel (**a**) were divided by the values on panel (**b**)]. Thylakoid membranes were incubated in 0 µM (black), 1 µM (red), 5 µM (blue) or 10 µM RB (green)

The detailed time course of *F*_v_/*F*_m_ changes show that PSII activity was gradually decreased in the light-exposed thylakoid membranes (Fig. 2a). Importantly, this effect showed a strong dependence on RB concentration. While in the absence of RB the thylakoids retained ca. 75% of their initial activity even after 30 min of light exposure, in the presence 10 µM RB a complete loss of *F*_v_/*F*_m_ occurred (Fig. 2a). The thylakoids which were treated with 1 and 5 μM RB showed an intermediate behavior. The *F*_v_/*F*_m_ values showed some decrease even in darkness. This effect was negligible in the absence of RB up to 30 min (95% of the initial activity remained), but was enhanced when RB was also present (80% remaining activity at 10 μM RB after 30 min) (Fig. 2b).

To compensate for the slow dark inactivation of PSII activity of thylakoids the F_v_/F_m_ data are also shown after normalization of the data obtained in the light to those obtained in the dark (Fig. 2c). This representation shows the extent of PSII activity loss, which is actually caused by externally generated ^1^O_2_.

### Histidine ameliorates the photodamaging effect of externally produced ^1^O_2_ in thylakoids

**Fig. 3:**
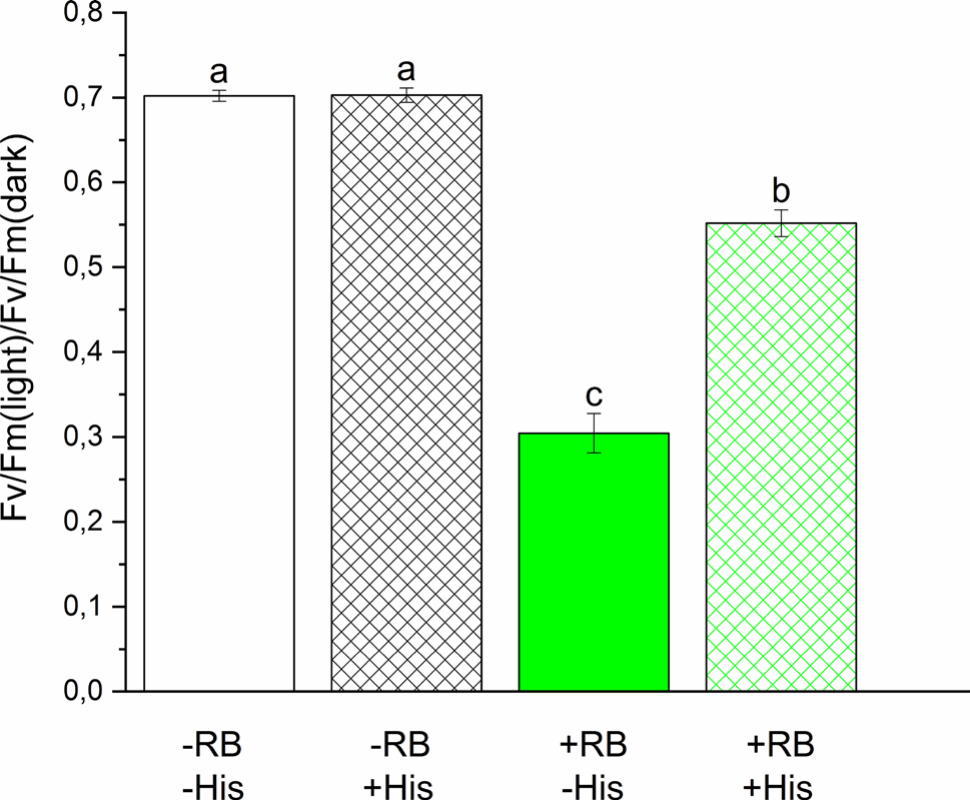
Effect of histidine on RB-induced loss of PSII activity. Thylakoids were illuminated for 60 min in the absence and presence of 5 μM RB as in Fig. 1, either without further addition, or in the presence 10 mM histidine. The *F*_v_/*F*_m_ values are shown after correction for the change that occurs during dark storage of samples. Different letters above the bars indicate significant differences (*p* < 0.05)

To check if the observed loss of PSII activity by illumination in the presence of RB is indeed related to ^1^O_2_-induced damage or not, His was also added to the thylakoid samples. His is a known chemical scavenger of ^1^O_2_ (Matheson and Lee 1979; Méndez-Hurtado et al. 2012), which can be used in photosynthetic systems without affecting electron transport (Rehman et al. 2013). The addition of 5 mM His together with 5 μM RB provided a significant protection against the loss of PSII activity when compared to the effect of RB addition alone (Fig. 3), confirming the involvement of ^1^O_2_ in PSII damage.

### Externally produced ^1^O_2_ accelerates photodamage of PSII in intact *Chlorella* cells

**Fig. 4:**
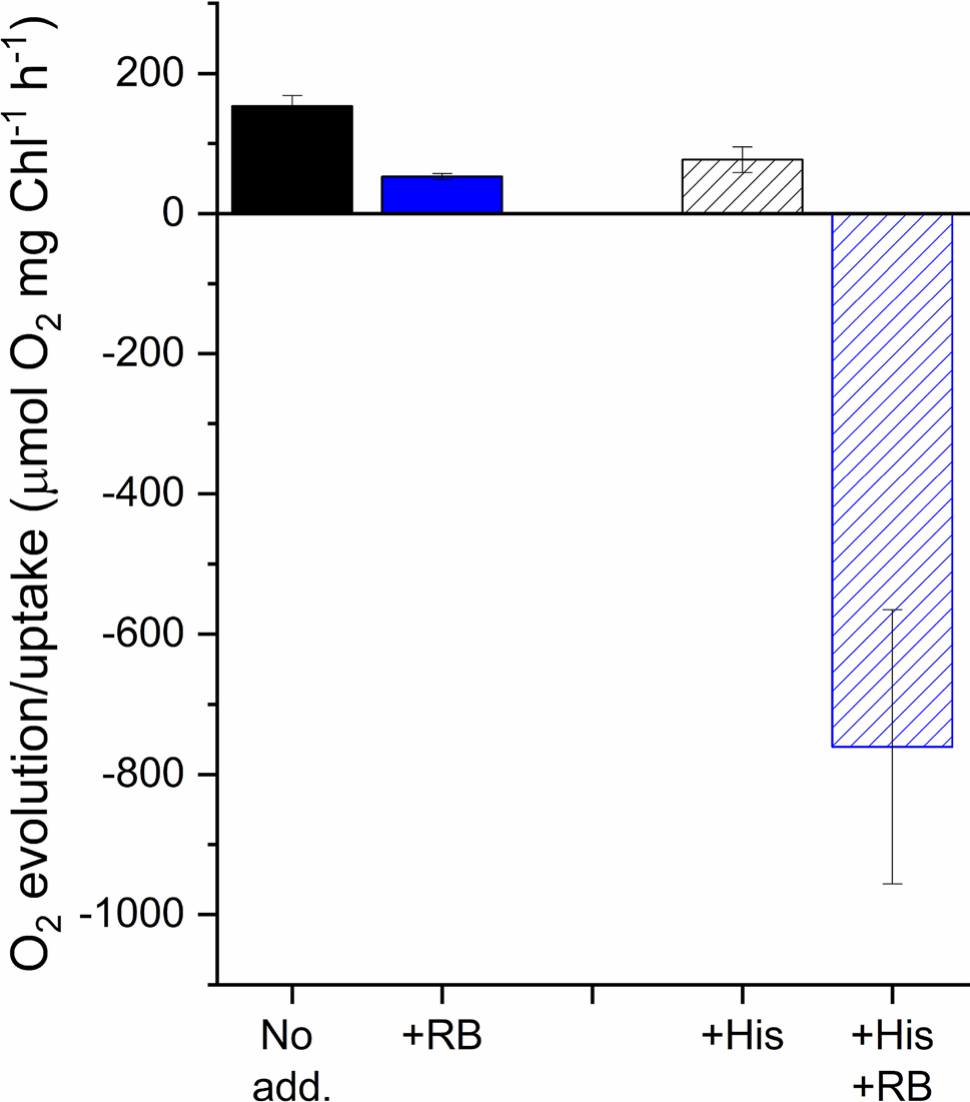
Oxygen evolution/uptake of Chlorella cells. Black, no treatment, blue, 1 μM RB treatment. Closed bars, no His, checked bars, in the presence of 5 mM His

To assess the effect of externally produced ^1^O_2_ in intact cells first we wanted to have an estimation of the ^1^O_2_ flux, which is produced by the illumination of RB. This was achieved by using the His-mediated chemical trapping method, which removes dissolved O_2_ from the medium as a results ^1^O_2_-induced oxidation of His, which results in easily measurable O_2_ uptake (Rehman et al. 2013). Using this approach we showed that the O_2_ evolution rate decreased in *Chlorella* cells in the presence of 5 mM His compared to control cells in which His was absent, showing a ca. 52.8 ± 4.2 μmol O_2_ mg Chl^−1^ h^−1^ rate of ^1^O_2_ production under the illumination conditions which were applied for the photoinhibition experiments. When *Chlorella* cells were illuminated in the presence of 1 μM RB the O_2_ evolution rate was significantly reduced due to chemical trapping of ^1^O_2_ by proteins and lipids in the cells. Addition of His together with RB resulted in a large O_2_ uptake (Fig. 4), which shows a ca. -760.8 ± 195.5 μmol O_2_ mg Chl^−1^ h^−1^ rate of ^1^O_2_ production in the presence of RB. This ^1^O_2_ production rate in the bulk medium is significantly higher than the ^1^O_2_ rate, which arises from the photosynthetic pigments, i.e. in the absence of RB. However, although RB has been shown to reach chloroplasts in intact leaves (Kovács et al. 2014), its local concentration is most likely lower in the chloroplasts of the *Chlorella* cells than in the bulk medium. Therefore, the ^1^O_2_ concentration in the vicinity of PSII is expected to be somewhere between the ambient level, produced by the photosynthetic pigments in the absence of RB, and the bulk level in the culture medium.

**Fig. 5:**
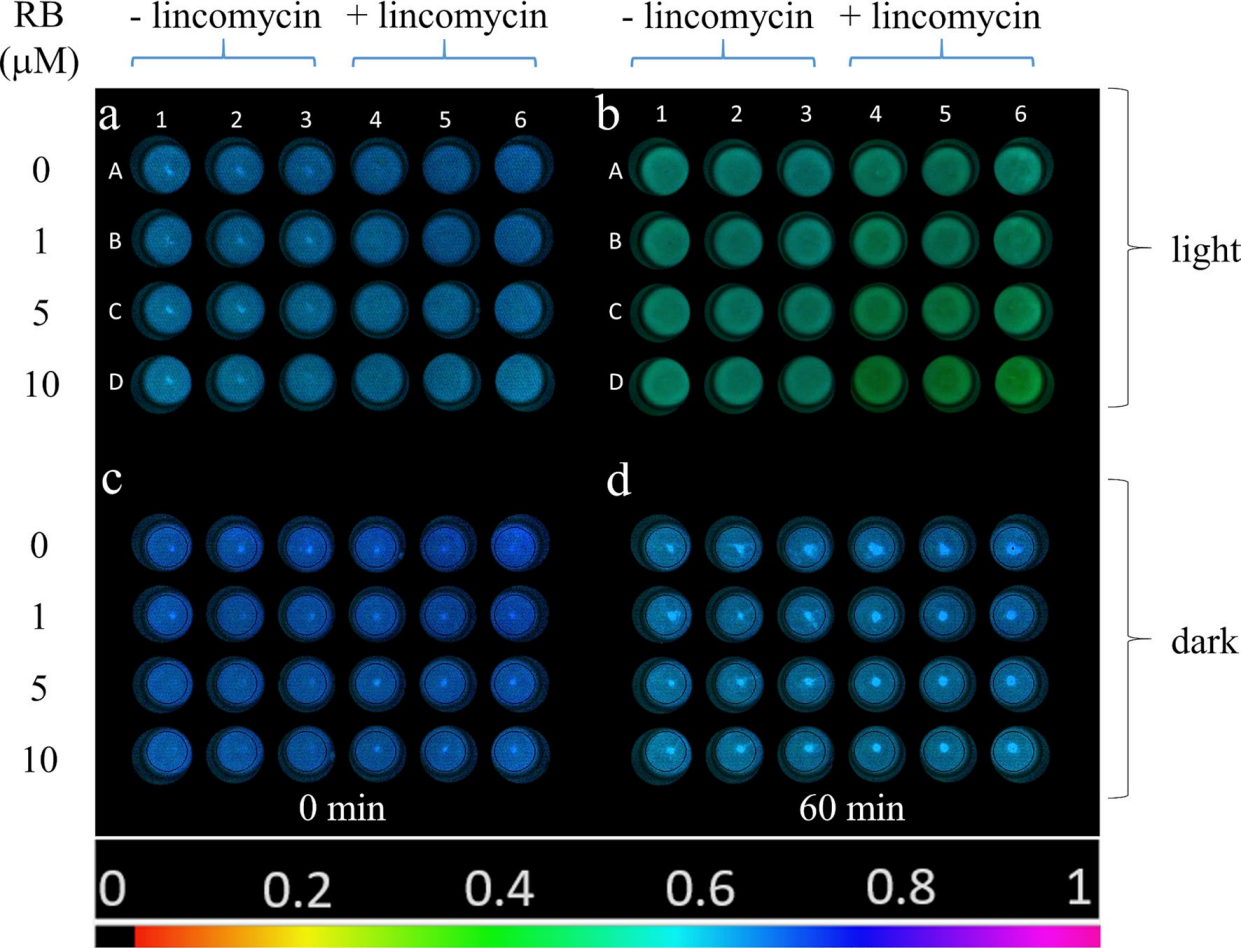
*F*_v_/*F*_m_ image of *Chlorella sorokiniana* cells in the 24 well plates illuminated with green + white light (**a**, **b**) or kept in darkness (**c**, **d**) at 0 min (**a**, **c**) and after 60 min (**b**, **d**). Wells A1-3: 0 µM RB, A4-6: 0 µM RB + lincomycin, B1-3: 1 µM RB, B4-6: 1 µM RB + lincomycin, C1-3: 5 µM RB, C4-6: 5 µM RB + lincomycin, D1-3: 10 µM RB, D4-6: 10 µM RB + lincomycin. The color bar indicates the relative intensity of *F*_v_/*F*_m_ (black: *F*_v_/*F*_m_ = 0, magenta: *F*_v_/*F*_m_ = 1)

To clarify the effect of externally generated ^1^O_2_ on PSII activity in vivo, intact *Chlorella* cells were incubated in light and in darkness in the presence of different concentrations of RB. In intact systems photodamage and repair of PSII proceeds in parallel. To separate these two processes, the experiments were performed both in the absence and presence of lincomycin, which blocks protein synthesis dependent PSII repair and thus allows the investigation of PSII photodamage without the effect of the ongoing repair. At the beginning of the experiment (*t* = 0 min) the cells exhibited similar *F*_v_/*F*_m_ values (0.63–0.65) in the entire plate, indicating that the RB treatment did not affect maximal PSII quantum yield (Fig. 5a). After 60 min light treatment, a RB-concentration dependent decrease in *F*_v_/*F*_m_ could be observed, which was more pronounced in the presence of lincomycin (Fig. 5b, well columns 4–6). When the cells were incubated in complete darkness only a minor decrease in *F*_v_/*F*_m_ was observed, which was independent of RB concentration (Fig. 5d).

**Fig. 6:**
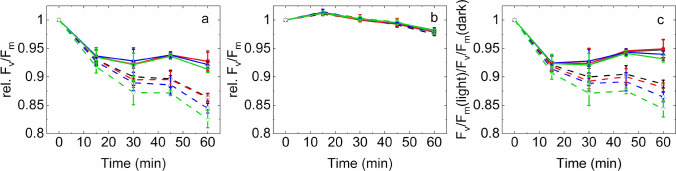
Effect of RB on the maximum quantum efficiency of PSII (*F*_v_/*F*_m_) of *Chlorella sorokiniana* cells treated with green-white light (240 μmol photons m^−2^ s^−1^) (**a**), or kept in dark (**b**) for the indicated time periods. Panel **c** represents the ratio of F_v_/F_m_ recorded during the light treatment and dark incubation experiment [*F*_v_/*F*_m_ values in panel (**a**) were divided by the values on panel (**b**)]. Cells were incubated in the presence of 0 µM (black), 1 µM (red), 5 µM (blue) or 10 µM (green) RB. Solid lines, cells with no lincomycin, dashed lines, cells in the presence of 300 μg/ml lincomycin

**Fig. 7:**
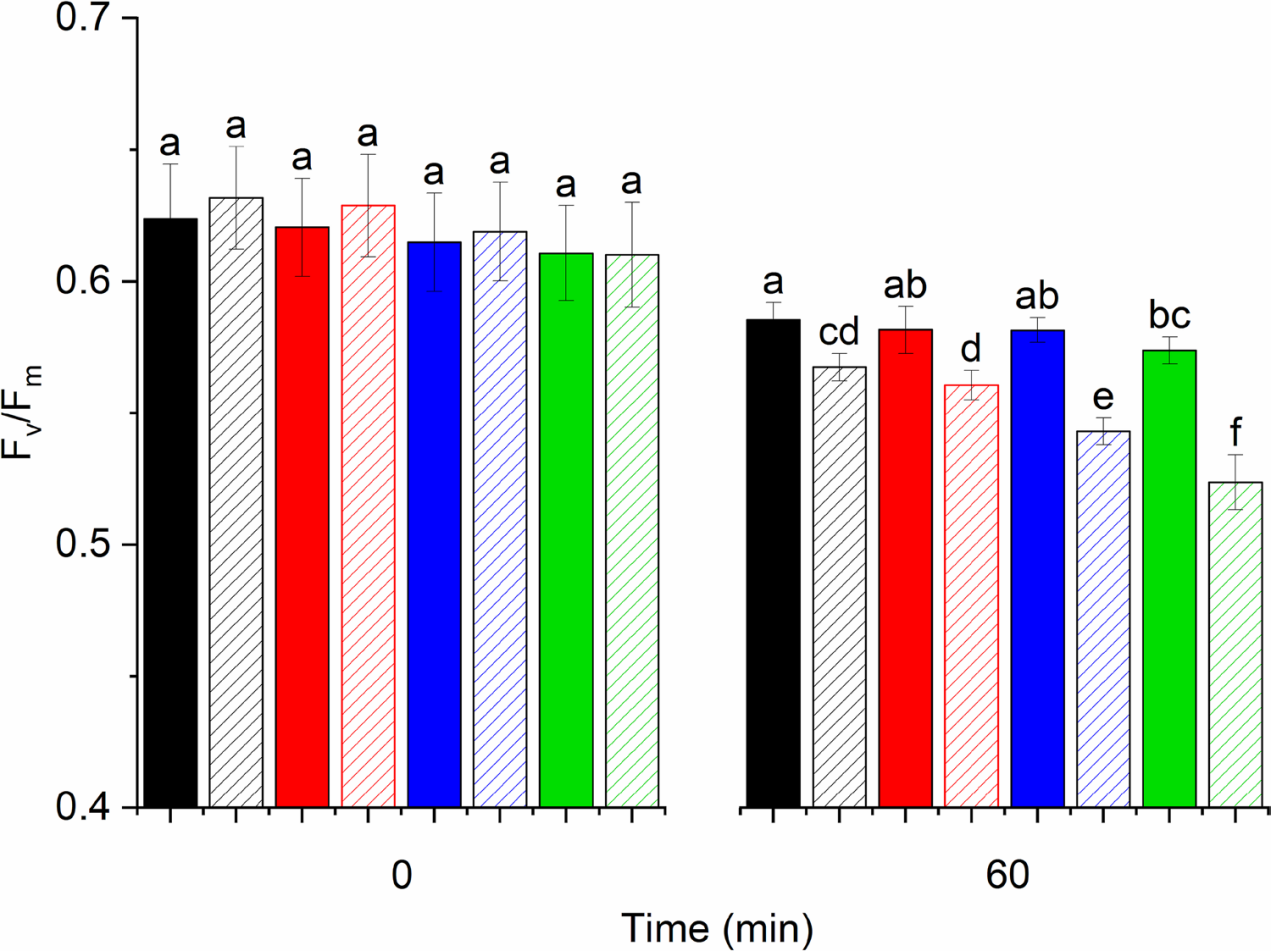
Maximum quantum efficiency of PSII (*F*_v_/*F*_m_) of *Chlorella sorokiniana* cells treated with green-white light (240 μmol photons m^−2^ s^−1^) in the presence of 0 µM (black), 1 µM (red), 5 µM (blue) or 10 µM (green) RB for the indicated time periods (*n* = 9). Closed bars, cells with no lincomycin, checkered bars, cells in the presence of 300 μg/ml lincomycin. Different letters above the bars indicate statistically different means, values sharing common letters are not significantly different from one another (*p* < 0.05)

The detailed time course of *F*_v_/*F*_m_ values shows that in the absence of lincomycin PSII activity loss showed a tendency to increase after 60 min light exposure in the presence of RB (Fig. 6a). However, this effect was statistically significant only after 60 min light exposure (see below, Fig. 7). The presence of lincomycin accelerated PSII damage, which became clearly visible already after 30 min (Fig. 6a). The enhancement of PSII photodamage showed a clear dependence on RB concentration. While 1 μM RB had practically no effect on *F*_v_/*F*_m_, 5 and 10 μM produced significantly larger *F*_v_/*F*_m_ loss after 30 min, than 0 or 1 μM RB (Fig. 6a). When *Chlorella* cells were kept in darkness the *F*_v_/*F*_m_ values showed a minor increase followed by a small decrease reaching ca. 97.5% of the initial PSII activity was kept after 0 min independent of the absence or presence of RB after 60 min (Fig. 6b).

To correct for the small changes of PSII activity in the dark the F_v_/F_m_ data are also shown after normalization of the data obtained in the light to those obtained in the dark (Fig. 6c). The changes in *F*_v_/*F*_m_ are relatively small in *Chlorella* cells as compared to those obtained in isolated thylakoids, when the same irradiance and timescale of treatment is applied (Fig. 2). However, after 60 min light treatment the enhanced decline of *F*_v_/*F*_m_ is obvious in the presence of RB, especially in the lincomycin treated cells (Fig. 6c). Although Fig. 6 clearly shows the tendency of cells experiencing larger PSII activity loss in the presence of higher RB concentrations, the extent of these changes was also analyzed for statistically significant differences. As shown in Fig. 7, illumination of the *Chlorella* cells in the absence of RB resulted in a significant loss of the maximal quantum yield of PSII after 60 min even in the absence of lincomycin, showing that despite the relatively low light intensity photoinhibition of PSII has occurred. The PSII activity loss was enhanced in the presence of lincomycin due to the lack of protein synthesis dependent repair. This effect was further enhanced when RB was present, as an external source of ^1^O_2_, and the extent of PSII activity loss was increasing practically linearly with increasing concentration of RB. In the absence of lincomycin there was a tendency of increased PSII activity loss in the presence of RB, but the effect was statistically significant only in case of 10 μM RB.

## Discussion

### Externally produced ^1^O_2_ is capable of damaging PSII both in isolated thylakoids and intact Chlorella cells

It is generally accepted that ^1^O_2_, as one of the highly reactive ROS forms, is involved in the process of photoinhibition﻿ (for reviews see Krieger-Liszkay 2005; Nishiyama et al. 2006; Vass 2011; Tyystjarvi 2013). However, the exact action mechanism of ^1^O_2_ is debated. The main topic of this debate is whether ^1^O_2_ can directly damage the PSII complex, or it can inhibit only the protein synthesis dependent repair of PSII. The idea that ^1^O_2_ is an elicitor of PSII photodamage is supported by various lines of experimental evidence. These findings demonstrate ^1^O_2_ production in light exposed isolated (Macpherson et al. 1993; Hideg et al. 1994a) and intact (Hideg et al. 1998; Rehman et al. 2013, 2016b) photosynthetic systems. In addition, the correlation between the rate of photodamage and the rate of ^1^O_2_ production has also been clearly shown (Rehman et al. 2013; Hakkila et al. 2013, 2014; Bersanini et al. 2014; Sedoud et al. 2014). The findings that higher ^1^O_2_ production rates are accompanied in general with higher PSII photodamage rates do not necessarily prove that ^1^O_2_ is the cause of PSII damage, since in principle it may happen that photodamaged PSII centers produce more ^1^O_2_ than the active ones. However, the effect of mutations that enhance or decrease photodamage in parallel with modifications of ^1^O_2_ production pathways in PSII, which suppress or enhance protective charge recombination routes, respectively (Fufezan et al. 2007; Rehman et al. 2013), provide strong support for the idea that ^1^O_2_ is indeed an agent that induces or mediates the damage of PSII. This idea is supported also by the correlation of extreme light tolerance and decreased ^1^O_2_ levels in *Chlorella ohadii* (Treves et al. 2016).

The idea that ^1^O_2_ is a direct damaging agent of the PSII complex was challenged on the basis experiments, in which no PSII activity loss was observed when *Synechocystis* PCC 6803 cells were illuminated in the presence of RB, as an external ^1^O_2_ source, as well as chloramphenicol as protein synthesis inhibitor (Nishiyama et al. 2001, 2004, 2006). In contrast to these findings infiltration of RB into lincomycin treated intact tobacco leaves accelerated the loss of PSII activity and of the D1 protein (Hideg et al. 2007; Kovács et al. 2014) supporting the idea that external ^1^O_2_ can damage the structure and function of PSII. However, the high concentration of RB which was applied in these leaf infiltration studies (1 mM and 100 μM, respectively) raised the possibility of unspecific damage by the high concentration of externally generated ^1^O_2_.

Our present data provides evidence that illumination in the presence of RB can indeed damage PSII activity even when relatively low concentrations of RB (1–10 μM) are used, and this damaging effect can be ameliorated in thylakoids by adding histidine, which is a chemical scavenger of ^1^O_2_ (Fig. 3). These demonstrate that externally generated ^1^O_2_ inactivates PSII and confirm the validity of earlier leaf infiltration studies in which much larger concentrations (100–1000 μM) of RB were used in (Hideg et al. 2007; Kovács et al. 2014). The damaging effect is obviously more substantial in the isolated thylakoids (Figs. 1 and 2) than in the intact *Chlorella* cells (Figs. 5–7), but even in the latter case the enhancement of PSII activity loss is statistically significant and increases with increasing RB concentrations, especially in the presence of lincomycin, which blocks protein synthesis dependent PSII repair (Fig. 7). ^1^O_2_ has a very short lifetime and travel distance in a cellular environment (Krasnovsky 1998; Skovsen et al. 2005), therefore it is unlikely that the ^1^O_2,_ which is produced outside the cells could reach the thylakoid embedded PSII complexes in the chloroplasts. The ability of RB to penetrate inside the cells and reach chloroplasts has been shown in tobacco leaves (Kovács et al. 2014). RB can also influence intracellular processes in *Synechocystis* 6803 (Nishiyama et al. 2004) and *Chlorella vulgaris* (Dall’Osto et al. 2019) and this is also the case in *Chlorella sorokiniana* used in the present study. The reason for the weaker inhibition of PSII in the intact *Chlorella* cells in comparison to the isolated thylakoids should be related to the lower local RB concentration inside the chloroplasts in the algal cells as compared to the bulk medium, and/or the presence of efficient ^1^O_2_ scavenging processes in the intact system. This idea is supported by the observation that even though a large amount of ^1^O_2_ was produced at 1 μM RB concentrations in the bulk medium (Fig. 4) PSII activity loss was significant only in the higher concentration range of 5–10 μM (Figs. 5, 6).

Similarly to the case of isolated thylakoids we have attempted to check the protective effect of histidine also in the *Chlorella* cells. However, the results were not conclusive (not shown) most likely due to the relatively small extent of PSII activity loss, which was observed as a result of illuminating *Chlorella* cells in the presence of RB (Figs. 6, 7). It is also possible that the penetration of histidine to the vicinity of PSII complexes inside the *Chlorella* cells, where it could exert its protective action, was limited. However, based on the strong protective effect of histidine in thylakoids, we can assume that histidine would ameliorate PSII activity loss in *Chlorella* as well, provided that it can reach the thylakoids inside the cells.

The loss of PSII activity by externally generated ^1^O_2_ is in full agreement with previous studies, which showed the impact of ^1^O_2_ in vitro can be related to the fragmentation of D1 protein, i.e. the scission of peptide bonds in the D1 protein (Lupínková and Komenda 2004; Miyao 1994), and consequently inactivation of electron transport (Mishra et al. 1994), and also with the degradation of the D1 protein during illumination of RB infiltrated leaves (Hideg et al. 2007; Kovács et al. 2014). In addition, mass spectrometry analysis of photodamaged PSII showed the presence of oxidized amino acid residues both at the acceptor and donor sides of the PSII reaction center complex, which demonstrates the damage of the D1 and D2 proteins by reactive oxygen species (Kale et al. 2017; Zhou et al. 2021).

### Reasons for contradiction with earlier data

Considering the contradiction of results in the present study concerning the damaging effect of externally produced ^1^O_2_ with those of earlier studies by Nishiyama and coworkers in which no effect of RB induced ^1^O_2_ on PSII damage rate was observed (Nishiyama et al. 2004) it is important to clarify the possible causes of this disagreement. There are four main differences of the experimental conditions in the present study and in the previous investigation (Nishiyama et al. 2004): (i) Isolated spinach thylakoids and *Chlorella sorokiniana* cells vs. the cyanobacterium *Synechocystis* PCC 6803. (ii) Relatively weak (240 µmol photons m^−2^ s^−1^) green dominated light to specifically excite RB without significant excitation of Chl vs. strong illumination by white light (1500 μmol photons m^−2^ s^−1^). (iii) Lincomycin vs. chloramphenicol as protein synthesis inhibitor in the present study. (iv) PSII activity was assessed here by Chl fluorescence imaging to determine *F*_v_/*F*_m_ vs. O_2_ evolution measurements by a Clark type oxygen electrode by Nishiyama et al. (Nishiyama et al. 2004). In the following section we discuss the significance of these differences.

#### Experimental objects

In order to study the effect of externally produced ^1^O_2_ on PSII activity it has to be made sure that the added photosensitizer, in this case RB, can reach the vicinity of the target, i.e. the PSII complex. This can be easily achieved in the aqueous suspension of isolated thylakoid membranes. Therefore, we choose thylakoids as one of our experimental object. The previous work (Nishiyama et al. 2004) was performed with the cyanobacterium *Synechocystis* 6803, which is undoubtedly a very useful and widely used model organism for photosynthesis and photoinhibition studies. However, the lack of RB-induced PSII damage might have been caused by a limited penetration of RB inside the *Synechocystis* cells limiting the amount of ^1^O_2_ in the vicinity of the thylakoid membranes. Therefore, we choose the green alga *Chlorella sorokiniana*, since penetration of RB into *Chlorella* cells has been demonstrated in the closely related *Chlorella vulgaris* species (Dall’Osto et al. 2019). Since the damaging effect of RB-mediated ^1^O_2_ production was clearly seen in both the isolated thylakoids (Figs. 1–3) and in the *Chlorella* cells (Figs. 5–7), one factor in the lack of detection of PSII activity loss in *Synechocystis* (Nishiyama et al. 2004) is probably the limited reach of RB into the vicinity of the thylakoid embedded PSII complexes in this cyanobacterium. It is of note that some ^1^O_2_ was certainly produced by illumination of RB in the *Synechocystis* cells as shown by the inhibition of psbA mRNA elongation, which is a crucial step of the D1 protein synthesis dependent PSII repair (Nishiyama et al. 2004). However, the solubility of RB in water is much better than in organic solvents, therefore the RB sensitized ^1^O_2_ can more easily reach the site of D1 synthesis, which takes place in ribosomes attached to the cytosolic surface of the thylakoid membrane (Tyystjarvi et al. 2001), than the hydrophobic inner parts of the thylakoids where most of the functional components of the PSII complex are located.

#### Illumination protocol

We used relatively low light intensity (240 μmol photons m^−2^ s^−1^) with a significant contribution in the green spectral range that excites specifically RB, without driving the photosynthetic electron transport. As consequence we could avoid strong photoinhibition by photosynthetically active light and the damaging effect to PSII was induced mostly by the RB generated ^1^O_2_. In contrast, Nishiyama et al. (Nishiyama et al. 2004) used very strong (1500 μmol m^−2^ s^−1^) visible light, which in itself induced extensive photodamage of PSII, and combined this strong illumination with RB addition. As a consequence, the effect of RB addition on the photodamage rate was most likely relatively small, which could be left unnoticed. Actually, green illumination, which selectively excites RB was also used in the work by Nishiyama et al. (Nishiyama et al. 2004), but only for assessing the ^1^O_2_ effect on the repair process (i.e. in the absence of protein synthesis inhibitor). It is a puzzle why this straightforward approach was not used for assessing the ^1^O_2_ effect on the photodamage rate (i.e. in the presence of protein synthesis inhibitor)?

#### Protein synthesis inhibitors

To separate the processes of photodamage and repair of PSII intact cells have to be treated with a protein synthesis inhibitor. In photoinhibition studies the most popular protein synthesis inhibitors are lincomycin and chloramphenicol (Kodru et al. 2020). Their application is based on the assumption that they are not interacting with photosynthetic electron transport processes. Unfortunately, this is not the case for chloramphenicol, which has been shown to act as electron acceptor in PSI and reduce oxygen to reactive superoxide (Okada et al. 1991). More recently it was shown that chloramphenicol accepts electrons not only from PSI, but also from PSII (Rehman et al. 2016a), and also that this effect artificially enhances PSII photodamage via superoxide production in intact *Synechocystis* cells if chloramphenicol is applied above 100 μg mL^−1^ concentration (Kodru et al. 2020). Therefore, in the present study lincomycin was used in contrast to the work by Nishiyama et al. in which 200 μg mL^−1^ chloramphenicol was applied (Nishiyama et al. 2004). Similarly, to the effect of strong light the presence of high chloramphenicol amount could enhance the photodamage rate in the earlier study (Nishiyama et al. 2004) decreasing the chance to detect the RB effect.

#### PSII activity measurements

In the presence of RB illumination of the samples produce a large amount of ^1^O_2_, which can chemically react with, i.e. oxidize, proteins, lipids and other organic material in its environment. This oxidation results in O_2_ uptake that decreases the apparent O_2_ evolution rate detected by the Clark type electrode (see the decreased O_2_ rate in the presence of 1 μM RB in Fig. 6). Since in the previous *Synechocystis* study (Nishiyama et al. 2004) 2 and 10 μM RB was used the modification of the O_2_ evolution rate must have been much larger than that we observed here, unless RB was washed out from the cells before the O_2_ evolution assay. Although there is no indication for such a precaution in the Nishiyama paper this was most likely the case, otherwise the O_2_ evolution rate must have been very small even in the non-photoinhibited cells, which is not noted in the paper. The main reason for using Chl fluorescence imaging method in our present study was that it made possible to perform all treatments and PSII activity measurements under exactly the same experimental conditions. In addition, the Chl fluorescence imaging method is not affected at all by the ^1^O_2_ induced O_2_ uptake problem. Therefore, using Chl fluorescence imaging vs. O_2_ evolution measurements does not seem to be a crucial factor in the contrasting results.

Based on the above comparison of experimental conditions our conclusion is that the lack of detecting the RB-induced enhancement of PSII photodamage rate in the previous study (Nishiyama et al. 2004) was most likely the consequence of multiple effects: (i) The limited ability of ^1^O_2_ produced by RB to reach the thylakoid embedded functional components of the PSII decreased the chance of detecting PSII activity loss. (ii) The application of strong visible light instead of selective excitation of RB for the photodamage experiments together with using high chloramphenicol concentration enhanced the photodamage rate, which could easily mask the relatively small enhancement of ^1^O_2_-induced PSII damage. In our study these technical problems were eliminated by selective excitation of RB and by the application of lincomycin as protein synthesis inhibitor, that does not interfere with PSII electron transport and made possibly the detection of ^1^O_2_-induced PSII activity loss not only in the isolated thylakoids, but also in the intact *Chlorella* cells.

## Conclusions

We provided clear experimental evidence that ^1^O_2_ when generated by the externally added photosensitizer RB induces the loss of PSII activity both in isolated thylakoids and intact *Chlorella* cells, which are incapable of PSII repair. These data demonstrate that ^1^O_2_ when produced by an external source, outside of the photosynthetic apparatus, is capable of direct inactivation of PSII. Our results also provide support for the idea that ^1^O_2_ could act as an inducer of photodamage in native photosynthetic systems when it is produced by chlorophylls and other pigments (Vass and Cser 2009), in agreement with the proposal that light absorption in the catalytic Mn cluster cannot be solely responsible for the inactivation of PSII (Ohnishi et al. 2005), and other, Mn-independent damage mechanisms should also be considered (Oguchi et al. 2009, 2011; He et al. 2015; Iermak et al. 2020).

## Data Availability

All data presented are available in the form of figures, and tables in the main text.

## Abbreviations

Chl: Chlorophyll
PSI: Photosystem I
PSII: Photosystem II
RB: Rose Bengal
